# Effect of Ampicillin, Streptomycin, Penicillin and Tetracycline on Metal Resistant and Non-Resistant *Staphylococcus aureus*

**DOI:** 10.3390/ijerph110303233

**Published:** 2014-03-19

**Authors:** Dagmar Chudobova, Simona Dostalova, Iva Blazkova, Petr Michalek, Branislav Ruttkay-Nedecky, Matej Sklenar, Lukas Nejdl, Jiri Kudr, Jaromir Gumulec, Katerina Tmejova, Marie Konecna, Marketa Vaculovicova, David Hynek, Michal Masarik, Jindrich Kynicky, Rene Kizek, Vojtech Adam

**Affiliations:** 1 Department of Chemistry and Biochemistry, Faculty of Agronomy, Mendel University in Brno, Zemedelska 1, Brno CZ-613 00, Czech Republic; 2 Central European Institute of Technology, Brno University of Technology, Technicka 3058/10, Brno CZ-616 00, Czech Republic; 3 Department of Pathological Physiology, Faculty of Medicine, Masaryk University, Komenskeho namesti 2, Brno CZ-662 43, Czech Republic; 4 Karel Englis College, Sujanovo nam. 356/1, Brno CZ-602 00, Czech Republic

**Keywords:** *S. aureus*, antimicrobial resistance, antibiotics, metal resistance, cross resistance, growth curves, inhibition concentrations, spectrophotometry

## Abstract

There is an arising and concerning issue in the field of bacterial resistance, which is confirmed by the number of deaths associated with drug-resistant bacterial infections. The aim of this study was to compare the effects of antibiotics on *Staphylococcus aureus* non-resistant strain and strains resistant to cadmium or lead ions. Metal resistant strains were created by the gradual addition of 2 mM solution of metal ions (cadmium or lead) to the *S. aureus* culture. An increasing antimicrobial effect of ampicillin, streptomycin, penicillin and tetracycline (0, 10, 25, 50, 75, 150, 225 and 300 µM) on the resistant strains was observed using a method of growth curves. A significant growth inhibition (compared to control) of cadmium resistant cells was observed in the presence of all the four different antibiotics. On the other hand, the addition of streptomycin and ampicillin did not inhibit the growth of lead resistant strain. Other antibiotics were still toxic to the bacterial cells. Significant differences in the morphology of cell walls were indicated by changes in the cell shape. Our data show that the presence of metal ions in the urban environment may contribute to the development of bacterial strain resistance to other substances including antibiotics, which would have an impact on public health.

## 1. Introduction

The seriousness of the problem of bacterial resistance is confirmed by the number of deaths associated with drug-resistant bacterial infections—only in the EU it affects 25,000 people a year [1]. Recently, with the discovery of multi-resistant strains in the broader community, public health officials have begun to realize the potential danger of the spread of these antibiotic resistant bacteria [2]. Antimicrobial resistance (AMR) is a resistance of microorganism to an antimicrobial medicine to which it was originally sensitive. Creation of a resistance effect is dependent on the genes located in plasmids that are infectious matter transferred to other cells, so the resistance between bacteria spreads rapidly. Resistant microorganisms are able to withstand an attack of antimicrobial medicines, so that standard treatments become ineffective and infections persist increasing the risk of spreading to others. The evolution of resistant strains is natural phenomenon that happens when microorganisms are exposed to antimicrobial drugs and resistant genes can be distributed between certain types of bacteria. The misuse of antimicrobial medicines accelerates this phenomenon.

The incidence of resistant microorganisms is monitored primarily in hospitals, but much higher risk is present in the soil and waters [3]. Resistance, generated in the external environment, is a natural development of every live system. Infections caused by resistant microorganisms often fail to respond to the standard treatment, resulting in prolonged illness and greater risk of death [4,5,6,7]. The death rate of patients with serious infections treated in hospitals is about twice as high compared to the patients with infections caused by non-resistant bacteria [8].

A high percentage of hospital-acquired infections are caused by highly resistant bacteria such as methicillin-resistant *Staphylococcus aureus* (MRSA) or multidrug-resistant enterococci Gram-negative bacteria. The general mechanisms of resistance are: (1) the limited penetration of antibiotics into the bacterial cell; (2) the change of the target structure (receptor); (3) metabolic changes within the bacterial cell, which prevents the effect of antibiotics on the target structures; and (4) enzymatic inhibition/inactivation of antibiotics [9,10,11,12,13,14]. Metal resistance of microbes is accomplished by intra- and extracellular mechanisms. Metals can be excreted via efflux transport systems, sequestering compounds of the cytosol can bind and detoxify metals inside the cell. The release of chelators into the extracellular milieu fixes the bound metals. The structure of the cell envelope is prone to bind large amounts of metals by sorption, thus preventing influx [15]. Newly discovered resistance mechanisms, such as enzymes produced by the bacteria that destroy last generation antibiotics, have emerged among several Gram-negative bacilli and have rapidly spread in many countries. This can render ineffective powerful antibiotics, which are often the last defence against multi-resistant strains of bacteria. This new resistance mechanism is associated with ordinary human pathogens (e.g., *Escherichia coli*) which cause common infections such as urinary tract infection.

The use of antibiotics and toxic metals should be considered carefully with the attention paid to the environmental impacts [16,17,18]. Heavy metals are toxic and can be harmful to organisms. For this reason, a number of organisms including bacteria develop processes which are able to withstand the effects of these pollutants [15]. Toxic metals in the environment can enrich the antibiotic multi-resistance property of bacteria [19]. Resende *et al.* [20] evaluated medically relevant bacteria in an aquaculture system and their susceptibility to antibiotics and toxic metals. Multidrug-resistant bacteria were also found to be tolerant to nickel, zinc, chromium and copper. In another study by Ji *et al.* [21], eight antibiotic resistance genes (ARG), seven heavy metals and six antibiotics were quantified in manures and soils collected from multiple feedlots in Shanghai (China). Overall, sulfonamide ARGs were more abundant than tetracycline ARGs. The significant positive correlations were found between some ARGs and typical heavy metals such as Cu, Zn and Hg. Similarly, in the study by Malik *et al.* [22], majority of the *Pseudomonas* isolates from water and soil exhibited resistance to multiple metals (Hg, Cd, Pb, Cu, Zn, Ni) and antibiotics (water—tetracycline, polymyxin B; soil— sulphadiazine, ampicillin and erythromycin) was presented [22].

With the appearance of antibiotic-resistant bacteria, increasing numbers of infections are causing huge losses to both economic concerns and social resources over recent decades, and this has become a global problem [2]. This study aimed on testing of non-resistant strain of bacterial culture wild type *S. aureus* and *S. aureus* strains resistant to heavy metals ions (cadmium = RCd or lead = RPb) exposed to different concentrations of four various antibiotics (ampicillin, streptomycin, penicillin and tetracycline). The antimicrobial activity of antibiotics on *S. aureus* bacterial culture was tested by the growth curves and the results were statistically evaluated.

## 2. Experimental Section

### 2.1. Cultivation of *S. aureus*

*S. aureus* (NCTC 8511) was obtained from the Czech Collection of Microorganisms, Faculty of Science, Masaryk University, Brno, Czech Republic. The strains were stored as a spore suspension in 20% (*v*/*v*) glycerol at −20 °C. Prior to use in this study, the strains were thawed and the glycerol was removed by washing with distilled water. The composition of cultivation medium was as follows: meat peptone 5 g/L, NaCl 5 g/L, bovine extract 1.5 g/L, yeast extract 1.5 g/L (HIMEDIA, Mumbai, India), sterilized MilliQ water with 18 MΩ. pH of the cultivation medium was adjusted at 7.4 before sterilization. The sterilization of the media was carried out at 121 °C for 30 min. in sterilizer (Tuttnauer 2450EL, Beit Shemesh, Israel). The prepared cultivation media were inoculated with bacterial culture into 25 mL Erlenmeyer flasks. After inoculation, the bacterial cultures were cultivated for 24 h on a shaker at 600 rpm and 37 °C. The bacterial culture, cultivated under these conditions, was diluted by cultivation medium to OD600 = 0.1 and used in the following experiments.

### 2.2. Preparation of Resistant Strains of S. aureus

The resistant strains of *S. aureus* have been developed in the laboratory. 2 mM basic solutions of heavy metals ions (Cd^2+^ and Pb^2+^) was added to non-resistant bacterial culture of *S. aureus*, cultivated in Luria Bertani medium. Low concentration (50 μM) of the metal ions in a medium was inoculated into the bacterial culture, and then the concentration of heavy metal ions was always increased by the concentration of 50 μM to the maximum possible dose for regeneration of *S. aureus*.

### 2.3. Chemicals, Preparation of Deionised Water and pH Measurement

Chemicals used in this study were purchased from Sigma-Aldrich (St. Louis, MO, USA) in ACS purity unless noted otherwise. The deionised water was prepared using Aqual 25 reverse osmosis equipment (Aqual, Brno, Czech Republic). The deionised water was further purified by using a Milli-Q Direct QUV apparatus equipped with a UV lamp. The resistance was 18 MΩ. The pH was measured using a WTW inoLab pH meter (Weilheim, Germany).

### 2.4. Heavy Metals Ions Preparation

Heavy metals used for the preparation of heavy metals ions have always been in the form of nitrates of these metals (Cd(NO_3_)_2_·4H_2_O and Pb(NO_3_)_2_) dissolved in 100 mL Milli-Q water and final concentration of these ions were always 2 mM.

### 2.5. The Microscopy of the Cells in Ambient Light

The inverted system microscope Olympus IX 71 (Tokyo, Japan) was used for the imaging of the cells. The cells in cultivation medium were pipetted (5 μL) on the microscope slide and covered by cover slip. The cover slip was placed on the sample and the immersion oil was used. The objective (PlanFLN; Mag. 100×; NA 1, 3; F.N. 26.5) and the magnification lens 1.6× were used, the total magnification was 1600×. The images were captured by Camera Olympus DP73 and processed by Stream Basic 1.7 Software, the images resolution was 4800 × 3600 pixels. The parameters were as follows: exposure time: 32 ms and ISO 200.

### 2.6. Determination of Growth Curves

The procedure for the evaluation of the antimicrobial effect of tested compounds and their combinations consisted in measuring of the absorbance using the apparatus Multiskan EX (Thermo Fisher Scientific, Bremen, Germany) and subsequent analysis in the form of growth curves. Non-resistant bacterial culture of *S. aureus* or resistant strain *S. aureus* to cadmium and lead ions were cultivated in LB medium for 24 h with shaking and was diluted with LB medium using Specord spectrophotometer 210 (Analytik, Jena, Germany) at a wavelength of 600 nm to absorbance 0.1 AU. On the microplate, these cultures were mixed with various concentrations of four types of antibiotics (ampicillin, streptomycin, penicillin and tetracycline) or *S. aureus* alone as a control for measurements. The concentrations of antibiotics were 0, 10, 25, 50, 75, 150, 225 and 300 μM. Total volume in the microplate wells was always 300 µL. The measurements were carried out at time 0, then each half-an hour for 24 h at 37 °C and a wavelength of 620 nm. The obtained values were analyzed in graphical form as growth curves for each variant individually.

### 2.7. Determination of Cadmium and Lead Ions by Atomic Absorption Spectrometry

The determination of cadmium and lead ions was carried out using 240FS Agilent Technologies atomic absorption spectrometer (Agilent, Santa Clara, CA, USA) with flame atomization. Cadmium was measured on the wavelength 228.8 nm with spectral bandwidth of 0.5 nm and lead it was 217.0 nm with spectral bandwidth of 1.0 nm. The mixture of air and acetylene was used for the flame atomization. Deuterium background correction was used and the signal was measured in integration mode for 3 s.

### 2.8. Interaction of Bacterial DNA Fragment of zntR and 16S Gene with Cadmium and Lead Ions

DNA fragments of zntR and 16S gene (16 μg/mL) were incubated with different concentrations of Cd(NO_3_)_2_ and Pb(NO_3_)_2_ in 1:1 ratio. The stock solutions of DNA fragment of zntR gene (16 µg/mL) were incubated with 0, 0.06, 0.24 and 0.95 mM Cd(II) and Cd(II) in water (ACS purity, Sigma-Aldrich). The same procedure was carried out for the DNA fragment of 16S gene. The samples were incubated for 60 min at 25 °C. After incubation, unbound cadmium and lead ions were removed using Amicon Ultra 3K centrifugal filter device (Millipore Corp., Billerica, MA, USA). After centrifugation at 14,000 rpm for 10 min at 25 °C (5417R Eppendorf, Hauppauge, NY, USA), the sample was complemented with water to the original volume (200 µL).

The spectra were recorded within the range from 220 to 420 nm using quartz cuvettes (1 cm, Hellma, Essex, UK) on a SPECORD 210 spectrophotometer (Analytik Jena, Germany) at 25 °C (Julabo, Sellbach, Germany). The spectra were recorded after 60 min of interaction. The denaturation of the complex of DNA with Cd(II) and Pb(II) was monitored spectrophotometrically using a SPECORD S600 spectrophotometer with a diode array detector (Analytik Jena). The sample was incubated for 3 min at increasing temperatures in a range from 25 to 90 °C and the absorbance was measured at 260 nm. The changes in absorbance spectra were recorded during denaturation. The absorption spectra were evaluated in the WinASPECT 2.2.7.0.

### 2.9. Determination of Metallothionein

The electrochemical detection was carried out using the method of differential pulse voltammetry (three electrodes involvement, working mercury drop electrode (HMDE), reference silver chloride electrode Ag/AgCl/3M KCl, and auxiliary carbon electrode) [23]. The analysed samples were deoxidized by argon bubbling for 120 s. As a supporting electrolyte, Brdicka solution (containing 1 mM Co(NH_3_)_6_Cl_3_ and 1 M ammoniacal buffer (NH_3_(*aq*) + NH_4_Cl, pH = 9.6)) was used. The supporting electrolyte was changed after each analysis of the sample. The parameters for the measurements were following: initial potential of −0.7 V, final potential of −1.75 V, the time interval 0.2 s, step potential 2 mV, amplitude −250 mV [24,25,26,27].

### 2.10. Expression of ZntR Gene and 16S Gene

#### 2.10.1. Isolation of RNA

Bacterial cultures (1 × 10^6^ of cells) were centrifuged at 300 rcf and 20 °C for 10 min and the pellets were resuspended in 100 µL of PBS buffer and 0.2 µL of RNase inhibitors. Thus the prepared solution was mixed with 100 µL of Tissue Lysis Buffer. The entire volume (200 µL) were pipetted into the sample tube, which is the component of the isolation kit MagNA Pure Compact RNA Isolation Kit (Roche, Basel, Schweiz), and inserted with other instruments on the appropriate place in the machine. In the second row of the machine, the vials with 20 µL of DNAase were inserted. Next steps were carried out according to the manufacturer’s instructions (“RNA Cell” protocol MagNA).

#### 2.10.2. Amplification of mRNA

The mRNA were converted to cDNA using PrimeScript One Step RT-PCR Kit Ver. 2 (TaKaRa, Mountain View, CA, USA). The reaction profile was as follows: initial denaturation at 94 °C for 2 min, 30 cycles of 94 °C for 30 s, 50 °C for 30 s and 72 °C for 1.5 min.

#### 2.10.3. Amplification of cDNA for zntR Gene

The *zntR* gene was amplified using polymerase chain reaction (PCR). The sequences of forward and reverse primers were 5'-GGATCCATGTCAGAACAATATTCAGA-3', and 5'-AAGCTTTTATAACCCACTTTCTTTAG-3' respectively. The final volume of the PCR reaction mixture was 25 μL containing 15.875 μL of sterile water, 2.5 μL of 1× *Taq* reaction buffer, 0.5 μL of 100 mM dNTP, 0.5 μL of forward primer, 0.5 μL of reverse primer and 0.125 μL of *Taq* DNA polymerase. The reaction profile was as follows: 30 cycles of 94 °C for 3 min, 50 °C for 1 min and 72 °C for 1 min and a final extension at 72 °C for 4 min. The amplification was carried out using Mastercycler ep realplex^4^ S (Eppendorf AG, Hamburg, Germany) and a 333 bp fragment was obtained.

#### 2.10.4. Amplification of cDNA for 16S Gene

The *16S* gene was amplified using polymerase chain reaction (PCR). The sequences of forward and reverse primers were 5'-GAGTTTGATCCTGGCTCAG-3' and 5'-GGTTACCTTGTTACGACTT-3' respectively. The final volume of the PCR reaction mixture was 25 μL containing 14.42 μL of sterile water, 2.5 μL of 1× *Taq* reaction buffer, 0.5 μL of 100 mM dNTP, 1.25 μL of forward primer, 1.25 μL of reverse primer and 0.085 μL of *Taq* DNA polymerase. The reaction profile was as follows: initial denaturation at 94 °C for 4 min, 30 cycles of 94 °C for 30 s, 52 °C for 30 s and 72 °C for 1.5 min and a final extension at 72 °C for 10 min. Finally, a 1500 bp fragment was obtained.

### 2.11. Statistical Analyses

The STATISTICA data analysis software system, version 10.0 (StatSoft, Tulsa, OK, USA) was used for data processing. The half-maximal concentrations (IC_50_) were calculated from logarithmic regression of sigmoidal dose-response curve. The general regression model was used to analyse differences between the combinations of compounds. To reveal differences between the cell lines, Turkey’s *post hoc* test within homogenous groups was employed. Unless noted otherwise, *p* < 0.05 was considered significant.

Besides applied antibiotics, the general regression model statistics were also used to analyse the effect of individual *S. aureus* strains. The data were natural logarithm-transformed and adjusted by the time variable using regression model. Consequently, Bonferroni post hoc test was employed to reveal the differences between *S. aureus* strains. Kruskal-Wallis and multiple comparisons of mean ranks test were used to compare the IC_50_ values based on MTT assay. Unless noted otherwise, *p*-level < 0.05 was considered significant. 

## 3. Results and Discussion

In the last decade, the number of infections caused by Gram-positive bacteria, resistant to formerly effective antibiotics, has increased significantly around the World. The phenomenon of multi-resistance considerably complicates the choice of antibiotics for infection treatment, which usually involves the combination of antibiotics in order to increase the efficiency [13]. Antibiotic drugs in particular groups are characterized by the same mechanism of action and very similar mechanism of resistance. The target of the antibiotics are mostly ribosomes where the antibiotics as an inhibitor of protein translocation are bound to the 50S subunit of the ribosome, stimulating the dislocation of peptide-tRNA from the ribosome during the elongation phase and induce rapid collapse of the polyribosomes [28].

Antimicrobial agents, their effects and resistance formation on various bacterial strains had very important impact on human and animal medicine. On the other hand, in view of global evolution theory, the resistance formation is only the next step at assimilation of live organism to the environment. The connection of effects from various antimicrobial agents leads to the formation of cross resistance. Concretely in our case, metal contamination functions as a selective agent in the proliferation of antibiotic resistance. This study is focused just on the investigation of metal (cadmium and lead) and antibiotic (PNC—penicillin, STR—streptomycin, AMP—ampicillin, TTC—tetracycline) resistance on bacteria *S. aureus* just because of one important fact that most antibiotics are readily degraded in the environment, but metals are not, and so can represent a long-term selective pressure.

### 3.1. Characterization on the Cellular Level

The testing was performed using bacterial culture *S. aureus* NCTC 8511 without the metal ions and two strains *S. aureus* NCTC 8511—RCd or RPb. The highest concentration of heavy metal ions in 24-h growing cell culture was 950 µM. For this experiment, the 950 µM resistant strains were recultivated for 24 h in clean medium. Selected antibiotics (penicillin, streptomycin, ampicillin and tetracycline) at various concentrations were applied to the tested bacterial strains.

#### 3.1.1. Morphological Characterization

**Figure 1:**
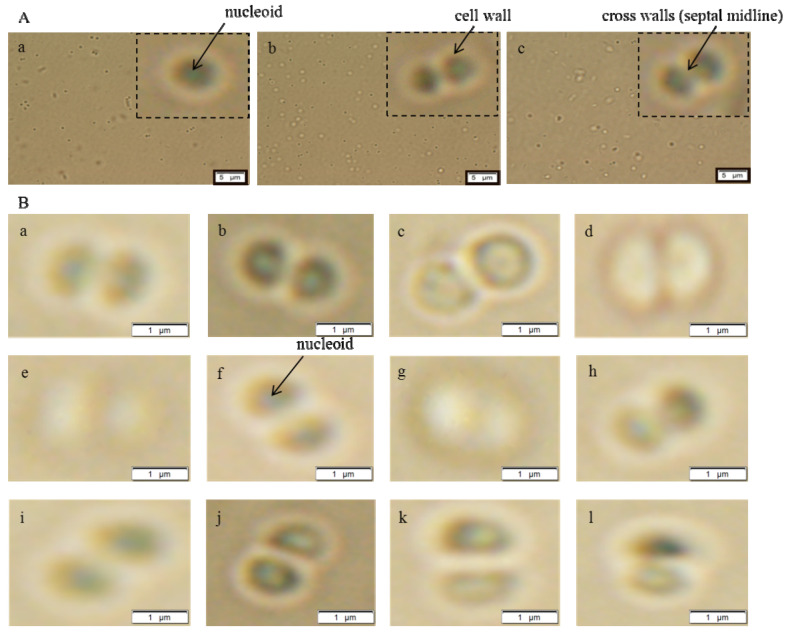
Images of *S. aureus* cells using microscopy in ambient light: (**A**) Micrographs of cells with resistance to metal ions (**a**) non-resistant strain of *S. aureus*; (**b**) RCd; (**c**) RPb. Parameters were as follows: Device: Microscopy; Volume: 5 μL; Zoom: 1600×; Ambient light; Exp.t.: 32.05 ms; ISO 200; Resolution: 4800 × 3600. (**B**) Micrographs of cells with resistance to metal ions after the application of antibiotics (50 µM): (**a**) penicillin; (**b**) streptomycin; (**c**) ampicillin; (**d**) tetracycline on non-resistant strain of *S. aureus*; (**e**) penicillin (**f**) streptomycin (**g**) ampicillin (**h**) tetracycline on RCd; or (**i**) penicillin (**j**) streptomycin (**k**) ampicillin (**l**) tetracycline on RPb.

Significant morphological changes were observed in the cells in terms of cell shapes. The presence of so called cross walls (septal midline) was observed (Figure 1). These inner transverse walls were formed due to the development of the resistance. Similar phenomenon is commonly observed in case of MRSA strains [29,30,31]. The above mentioned morphological changes were observed in both, metal resistant strains and control strain, after application of antibiotics. The detected phenomenon was the evidence of developing multi-resistance due to the fact that characteristic morphological hallmarks were present after addition of metal ions as well as antibiotics. Cell morphology was not influenced by the antibiotic administration due to the fact that the morphology was changed already by application of metal ions.

#### 3.1.2. Determination of Antimicrobial Activity

The mechanism of metal toxicity in the cell is determined by the interaction of the specific metals with a specific biological species [32]. Some metals like cobalt, copper, nickel and zinc are essential for many cellular processes in bacteria. However, higher concentrations of these can be cytotoxic. Other heavy metals, including lead, cadmium, mercury, silver and chromium have unknown beneficial effects on bacterial cells and are toxic even at low concentrations [33]. Metals enter into the cells in two ways [34]. The first way is mediated through non-specific transporters. The second way, substrate specific transport, is slower and often uses ATP hydrolysis as the energy source [35]. The heavy metal resistance in bacteria is connected with various possible mechanisms as follows: (a) exclusion of the metal by a permeability barrier; (b) exclusion by active export of the metal from the cell; (c) intracellular physical sequestration of metal binding proteins or other ligands to prevent it from damaging the metal-sensitive cellular targets; (d) extracellular sequestration and (e) transformation and detoxification [36].

Cadmium resistance is supported mainly by efflux mechanisms. *S. aureus* had cadmium resistance through an efflux mechanism consisting of the P-type ATPase transport system [34]. P-type ATPases are a metal transporting group of proteins involved in the transport of heavy metals across biological membranes. P-type ATPase consists of a single, large catalytic monomer of 70–200 kDa. The energy released by the removal of the γ-phosphate from ATP is coupled to the translocation of an ion across biological membranes. The direction of transport of P-type ATPases is mostly to the periplasm without further transport from the periplasm to the outside. These metal transporters prevent the over accumulation of highly toxic and reactive ions. The substrates *in vivo* are likely metal-thiolate complexes rather than the free metals. P-type ATPases can be divided into two subgroups: (I) Cu(I)/Ag(I)-translocating ATPases, e.g., *copA* in *Enterococcus hirae*, *Helicobacter pylori*, *Escherichia coli* and (II) Zn(II)/Cd(II)/Pb(II)-translocating ATPases, e.g., *zntA* in *E. coli* and *cadA* in *S. aureus* plasmid, pI258 [34,37].

Resistance to lead in *S. aureus* is probably mainly caused by the same *cadA* gene which encodes multipurpose P-type ATPase [38,39], but in addition to the membrane transport pumps (that remove metal ions from the bacterial cell), binding factors (detoxifying metals by sequestration) are also involved in tolerance to heavy metal ions in some bacteria. Intracellular bioaccumulation is usually connected with metallothioneins which play an important role in immobilization of toxic heavy metals, thereby protecting bacterial metabolic processes catalysed by enzymes [35]. Sequestration is also a detoxification mechanism for lead ions. *S. aureus* uses intra- and extracellular binding of lead to avoid toxicity of free lead ions by precipitating this element as a phosphate salt [40].

The synergic effect of antibiotics on RCd, RPb and control strain was investigated in this study. Antibiotics are one of the most commonly used drugs causing the resistance of the organism. Significant impact of the drug on the bacterial cell was observed after exposition of the cells with the existing heavy metal ions, resistance to the antibiotics. It can be expected that the mechanism of antibiotic action differs from metal ions and they do not overlap [10,41].

**Figure 2:**
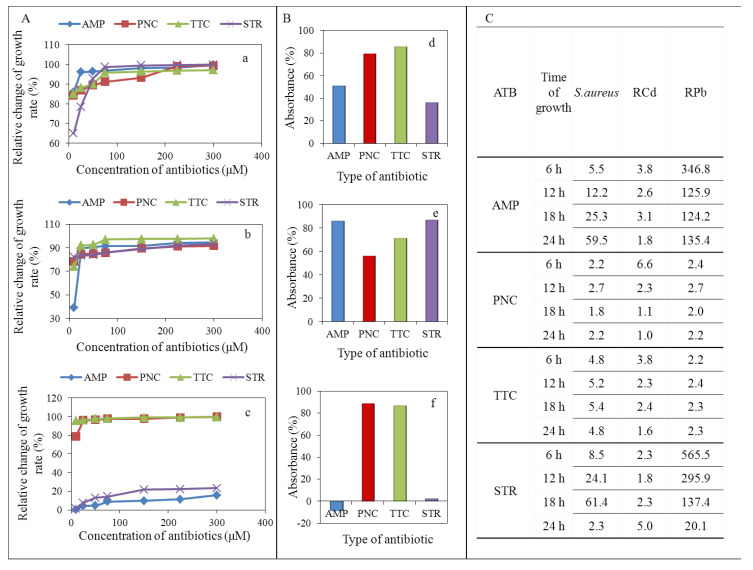
Spectrophotometric analysis of wild type *S. aureus* or *S. aureus* strains resistant to 950 µM concentration of heavy metal ions after application of various concentration of antibiotics (0, 10, 25, 50, 75, 150, 225 and 300 µM): (**A**) Relative change of growth rate as the relative difference between *S.aureus* strain without antibiotic and individual dose of antibiotic (related to the value obtained without antibiotic) in first 6 h of measurement in strains of *S. aureus* (%): (**a**) non-resistant *S. aureus*; (**b**) RCd; (**c**) RPb. (**B**) The cumulative effect of different types of antibiotics (%) on: (**d**) non-resistant *S. aureus*; (**e**) RCd; (**f**) RPb. All values were deducted from the non-resistant *S. aureus* (100%). (**C**) Inhibitory concentrations of tested antibiotics. AMP—ampicillin, PNC—penicillin, TTC—tetracycline, STR—streptomycin.

In the case of penicillin and tetracycline, the effect was manifested by growth inhibition in first 6 h of measurement in all tested strains, control strain (Figure 2Aa), RCd (Figure 2Ab) and RPb (Figure 2Ac). The decrease of growth in comparison to the control group was observed already after addition of 10 µM of penicillin and tetracycline. Further, the increase of penicillin and tetracycline concentrations influenced the final effect only slightly (Figure 2A).

After addition of ampicillin and streptomycin, decline in RPb growth was not observed even after application of 300 µM (Figure 2Ac). The results were also confirmed by deduction of the absorbance in 24 h of measurement (Figure 2B). Growth inhibition was not observed only in the strain resistant to the action of lead ions (RPb) after application of concentration range of streptomycin and ampicillin (Figure 2Ac,Bf).

The inhibition, expressed as inhibitory concentrations (IC_50_) values after first 6 h, confirmed the effects of tested antibiotics (Figure 2C). Figure 2C presents statistically evaluated values of IC_50_. This value is expressed as a concentration that causes 50% growth inhibition of test strain, in our case, non-resistant and resistant strains of *S. aureus*. The table shows the lowest IC_50_ values, determined in all strains tested after the application of penicillin and tetracycline. A small increase in values of IC_50_ was determined for the control strain and RCd after the application of ampicillin and streptomycin. The highest IC_50_ values were recorded for RPb after the application of ampicillin and streptomycin when diametrically diverging values was achieved. These indicate multi-resistance of RPb against the effect of these antibiotics.

**Figure 3:**
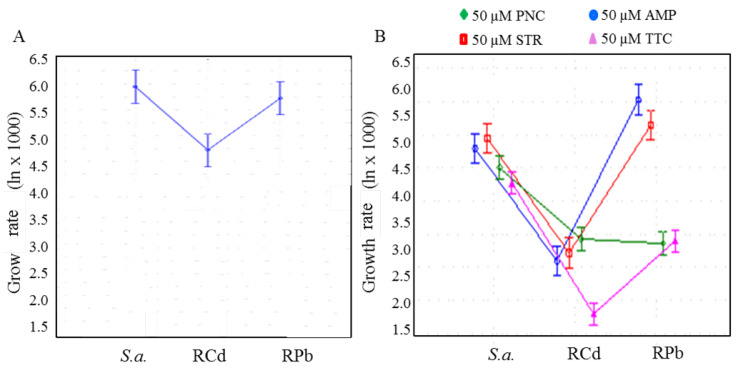
(**A**) Statistical evaluation of the cell culture behaviour at zero concentration of antibiotics, ANOVA F(8, 274) = 24.333, *p* < 0.001. (**B**) Comparison of growth rate at 50 µM concentration in each group for all antibiotics. ANOVA F(8, 270) = 65.860, *p* < 0.001. Data displayed as mean natural logarithm of residuals and 95 % confidence intervals. *S. a.*—*S. aureus* non-resistant strain, RCd, RPb. PNC—penicillin, STR—streptomycin, AMP—ampicillin, TTC—tetracycline.

After adjustment of the effect of time, growth rate was analysed on natural logarithm-transformed data. Significant differences between the resistant and sensitive group was determined, F(8, 274) = 24.33, *p* < 0.001 (Figure 3). To reveal the differences between the groups, Bonferroni *post hoc* test was employed: RCd expressed at lower growth rate and RPb expressed at higher growth rate were compared to sensitive strain (AMP, STR) and lower growth rate (PNC, TTC) at *p*-level < 0.05 (Table 1).

For penicillin, RCd strain expressed at lower growth rate and RPb cells expressed even at lower growth rate compared to sensitive strain at *p*-level < 0.05. For streptomycin, RCd strain expressed at lower growth rate and RPb cells expressed at higher growth rate compared to sensitive strain at *p*-level < 0.05. For ampicillin, RCd strain showed lower growth rate and RPb cells showed higher growth rate compared to sensitive strain at *p*-level < 0.05. For tetracycline, RPb strain expressed lower at growth rate and RCd cells expressed even at lower growth rate compared to sensitive strain at *p*-level < 0.05. Strain resistant to cadmium ions are generally characterized by lower growth rate after all tested antibiotics individually.

**Table 1:** Growth rate after application of 50 µM concentration of antibiotic in *S. aureus* non-resistant strain and strains resistant to cadmium and lead. Stars in columns indicate difference at *p*-level < 0.05 using Bonferroni *post hoc* test for homogenous groups.

Antibiotics	*S. aureus* strains	N	Absorbance (mean ± S.D × 10^3^)	Group different at *p* < 0.05
**Penicillin** (50 µM)				
	RPb	48	26.01 ± 0.76	****
	RCd	46	28.78 ± 0.9	****
	*S. a.*	48	90.81 ± 0.93	****
**Streptomycin** (50 µM)				
	RCd	46	21.83 ± 0.22	****
	*S. a.*	48	121.62 ± 1.26	****
	RPb	48	254.09 ± 0.83	****
**Ampicillin** (50 µM)				
	RCd	46	23.33 ± 3.21	****
	*S. a.*	48	175.22 ± 4.95	****
	RPb	48	323.05 ± 5.15	****
**Tetracycline** (50 µM)				
	RCd	46	9.86 ± 0.83	****
	RPb	48	30.04 ± 0.35	****
	*S. a.*	48	71.91 ± 0.58	****

The cross resistance is caused by the common presence of metal ions and antibiotics and represents a great problem. It is assumed that the presence of metal ions affects the antibiotic resistance through the two basic mechanisms as follows: (I) co-resistance (different resistance determinants present on the same genetic element) and (II) cross-resistance (the same genetic determinant responsible for resistance to antibiotics and metals) [42]. Co-resistance occurs when the genes, specifying resistant phenotypes, are located together on the same genetic element such as a plasmid. In contrast, the cross resistance occurs when different antimicrobial agents attack the same target. The result is development of resistance to one antibacterial agent accompanied by resistance to another. Considering the fact that structurally dissimilar compounds can use the same efflux mechanism, one may suggest that this can serve as an example of cross-resistance [42,43].

Resistance to antibiotics is usually achieved using four main strategies: (i) reduction of membrane permeability to antibiotics; (ii) drug inactivation; (iii) rapid efflux of the antibiotic; and (iv) mutation of cellular target(s) [42]. These mechanisms are predicted by chromosomal or mobile genetic elements (plasmids).

Penicillin and ampicillin belong to the group of β-lactam antibiotics where ampicillin is in the aminopenicillins subgroup. These antibiotics produce a bactericidal effect by inhibiting the membrane-bound enzymes responsible for catalysing vital stages in the biosynthesis of the cell wall. Such inhibition is the direct result of the covalent binding of the antibiotic to one or more penicillin-sensitive enzymes, termed penicillin-binding proteins (PBPs). The penicillin resistant bacteria produce an extracellular β-lactamase which inactivates antibiotics through hydrolysis of the β-lactam ring [44,45].

The β-lactamase structural gene (*blaZ*) is present on Tn*552-*like transposons [46]. These elements are located on β-lactamase plasmids which exhibit resistance to other antimicrobial agents, in particular heavy metal ions. There are four subgroups of these lactamase. The most famous and well described plasmids types are pI524 (encodes resistance to inorganic ions and organomercurials in addition to β-lactamase production), pI258 (except metal ion resistance carries the erythromycin resistance transposon Tn*551*) [46], and pSK23 which encodes metal ion resistance too. Tn*552*-like elements have been found at various sites in naturally occurring plasmids such as pSK4 and pSK1. The other locations of Tn*55*2-like transposons are chromosomes [45].

Tetracycline inhibits protein synthesis by binding to the 30S ribosomal subunit and preventing association of aminoacyl-tRNA with its acceptor site [47]. Two mechanisms of tetracycline resistance have been identified in *S. aureus*; active efflux via *tetA*(*K*) and *tetA*(*L*) and ribosomal protection via *tetA*(*M*) [45,47]. The closely related TetA(*K*) and TetA(*L*) efflux proteins belong to the major facilitator superfamily. In most cases, tetracycline efflux in *S. aureus* strains is mediated by *tetA*(*K*), which is commonly carried by plasmid pT181. This plasmid could be integrated into Type III SCC*mec* elements of chromosome and thus chromosomally encoded resistance. Resistance to tetracycline can also be mediated by mutations that cause increased expression of various chromosomally encoded efflux pumps, such as Tet38 [45,47].

Streptomycin exhibited resistance due to chromosomal mutations, affecting ribosome affinity [48]. Low-level resistance was usually indicative of small plasmids, such as pS194, which carries streptomycin adenyltransferase-encoding gene *str*. Chromosomal segment Tn*5405* is responsible forstreptomycin resistance through gene aphC [45,49].

From the presented summary of individual antibiotics resistance mechanisms, it is obvious that these mechanisms are predicted by the chromosomal and mobile genetic elements (plasmids, transposons). The above presented results show that the lead resistant *S. aureus* is resistant to ampicillin and streptomycin also. On the other hand, the cadmium resistant *S. aureus* shows no resistance to ampicillin and streptomycin. When these results were compared to above descripted mechanisms of antibiotic resistance, it seems that especially the role of transposons would be critical. The resistance of penicillin and tetracycline is related, among other things, to the transposons. In contrast, the streptomycin resistance is not connected with transposons. Similar results were published elsewhere [44]. The linkage of the metal ions (cadmium and lead) and antibiotic resistance is obvious at β-lactam antibiotics where the creation of co-resistance is offered due to the same location of genes related to various antimicrobial agents. The genes responsible for metal and antibiotic resistance located on plasmid pI258 are probably the main reason of cross resistance formation. But the behaviour of ampicillin is unknown. Its resistance, according to our theory, should not be related with transposons. But the general theory in literature for β-lactam antibiotics claims the contrary. No specific study according to the ampicillin resistance in *S. aureus* has been published yet. Comparison of the resulted data from different studies of toxic metal tolerance among bacteria is very complicated because of lack of technical standards in these experimental designs.

### 3.2. Characterization on the Molecular Level

The resistance to metal ions in *S. aureus* can be formed due to various mechanisms as follows: extracellular accumulation, sequestration by metallothioneins, intracellular physical sequestration, or efflux-based [36]. The efflux based mechanism is mainly supported by the occurrence of the *cadC* system present on a plasmid and the *znt* system present on the chromosome [50,51,52]. It was reported that *zntA* gene encodes a transmembrane structural protein, responsible for the efflux of zinc and cobalt ions in *S. aureus* [36]. In *E. coli* Znt efflux protein can also be activated by cadmium, lead and silver, but not by copper [36,53]. A chromosomally encoded *znt* operon in *S. aureus* consists of two consecutive putative genes designated as *zntR* and *zntA*. The structural gene *zntA* encodes a transmembrane protein that facilitates extrusion of zinc and cobalt ions, whereas the regulatory gene *zntR* encodes a putative regulatory protein that controls the expression of *znt* operon.

ZntR protein, encoded by *zntR* gene, regulates *zntA* and belongs to the family of regulators which introduce changes in the DNA conformation, which apparently make the promoter a better substrate for RNA polymerase [50]. It acts as a direct Zn sensor and catalyses transcriptional activation of zinc efflux gene.

One of the components of small subunit of prokaryotic ribosomes is 16S ribosomal RNA, conferred by 16S rDNA. This gene is about 1500 bp in length in *S. aureus* and it is often used in phylogenetic studies due to its hypervariable regions, useful for identification of bacteria (species or genera) [54].

**Figure 4:**
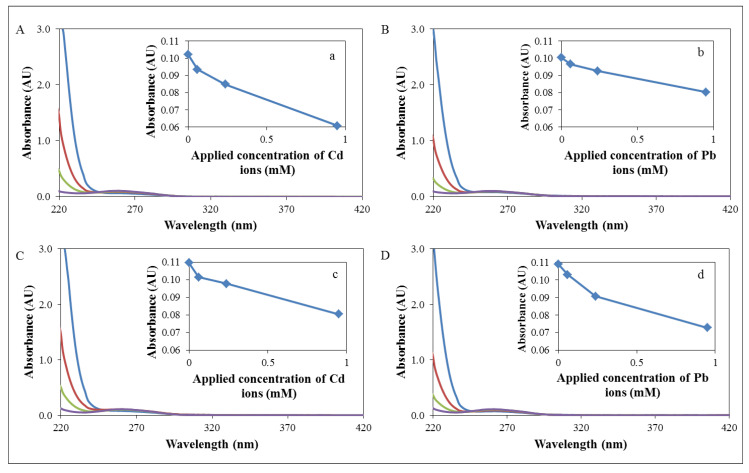
Interaction of bacterial DNA fragment with different concentrations of heavy metal ions (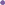 0, 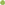 60, 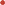 240 and 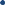 950 μM): (**A**) Interaction of 16S gene with cadmium ions, (**a**) decrease of absorbance of 16S gene with cadmium ions in 260 nm. (**B**) Interaction of 16S gene with lead ions, (**b**) decrease of absorbance of 16S gene with lead ions in 260 nm. (**C**) Interaction of *zntR* gene with cadmium ions, (**c**) decrease of absorbance of *zntR* gene with cadmium ions in 260 nm. (**D**) Interaction of *zntR* gene with lead ions, (**d**) decrease of absorbance of *zntR* gene with lead ions in 260 nm.

To confirm the simplified penetration of heavy metal ions into the cellular DNA, the interaction of cadmium and lead ions with a bacterial DNA fragment *zntR* and 16S was studied. This phenomenon was studied by spectrometric methods, particularly by absorbance measurement (Figure 4). The presence of heavy metal ions in DNA was manifested by a significant decrease of records for each concentration in comparison with a control sample. These results confirm the easy interaction of cadmium and lead ions with bacterial DNA.

**Figure 5:**
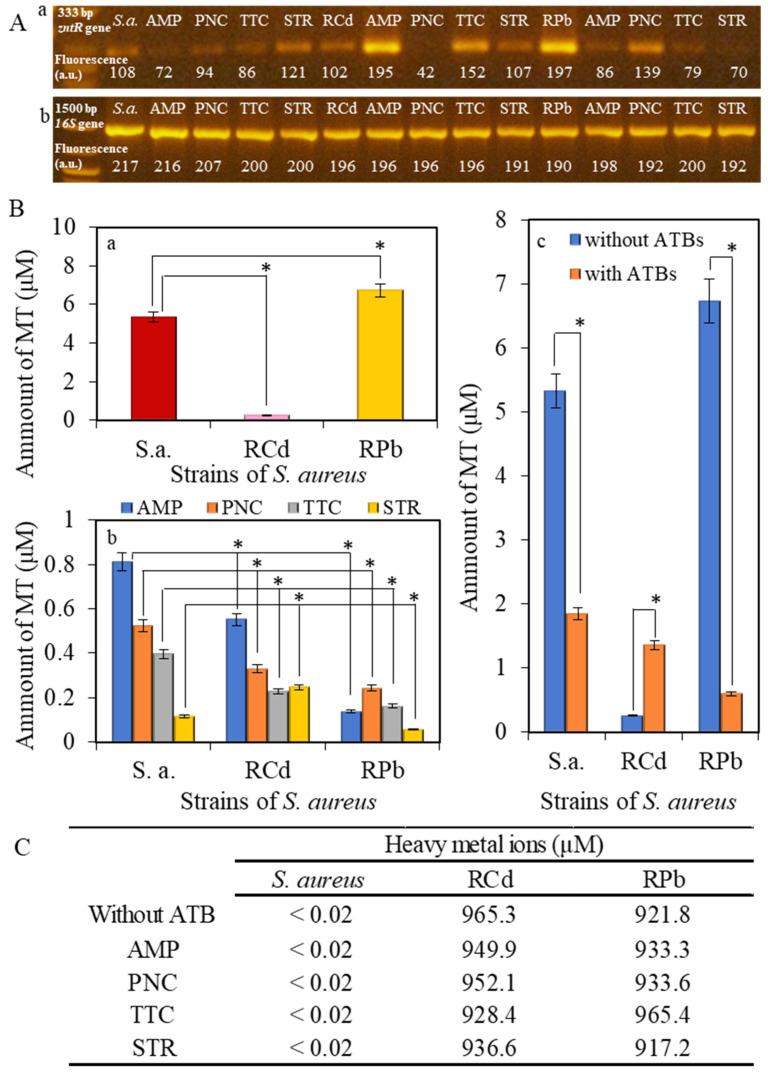
Electrochemical analysis of the non-resistant *S. aureus* or RCd or RPb: (**A**) gene expression with and without the application of antibiotics, (**a**) expression of *zntR* gene, (**b**) expression of 16S gene. (**B**) Determination of the amount of metallothionein (MT) in (**a**) *S. aureus*, RCd or RPb; and (**b**) *S. aureus*, RCd or RPb after application of 50 µM concentration of antibiotics; (**c**) comparison of the values of metallothionein in strains without application and after application of antibiotics. Presented values for ATBs are the sum of individual values for every antibiotic. All data represent mean ± s.d. NS, not significant, * *p* < 0.05, (**C**) Comparison of applied concentration of heavy metal ions (950 µM) with measured concentration using atomic absorption spectrometry.

The family of genes of the *S. aureus* bacterial strain is represented by *zntA* and *zntR* genes. These genes form open reading frames in the chromosomal fragment with the length of 3.2 kb, which confers resistance to heavy metal ions [55]. The genes *zntA* and *zntR* share the same promoter and are transcribed together [45]. In this work, we used the reverse transcription and amplification of fragment of the length of 333 bp [50] in the control strain of *S. aureus* NCTC 8511 and RCd or RPb and also in these strains after application of 50 µM concentration of antibiotics (Figure 5A).

The fluorescence intensity of the amplified fragment varies in dependence on the antibiotic type. In the RPb strains, the *zntR* gene’s expression was higher than that of control *S. aureus*. In contrast, in the RCd, the expression of the *zntR* gene was similar to the control *S. aureus* strain. In the RCd strain, after the addition of AMP and TTC, the expression of the *zntR* gene was significantly increased compared to the RCd without antibiotics. The most significant increase was observed after the application of ampicillin (Figure 5A(a)). The highest expression of the *zntR* gene was observed in the RPb strain without any addition of antibiotics. After the addition of antibiotics, the intensity of the expression was reduced (Figure 5A(a)). The expression of the *16S* gene (always present in bacteria) confirmed the presence of bacteria strain in the samples. This presence was independent of both on applied metal and antibiotics; therefore, the fluorescence intensity was constant (Figure 5A(b)).

Bacterial genome contains genes coding different proteins providing the tolerance to the heavy metal: metallo-regulatory genes [56]. These genes are usually from the family *ArsR/SmtB*. The sequences of these genes are usually homologous between the members of this family, but may differ in their active sites for binding metal [57]. Based on limited sequence homology, SmtB appears to be a member of a family of bacterial metalloregulatory proteins, including the ArsR proteins that are repressors of the arsenic-resistance operons and the CadC proteins which are the cadmium responsive repressors of expression of the cadmium efflux ATPase [57]. The SmtB, ArsR and CadC proteins contain conserved cysteine residues associated with the N-terminal extremity of putative DNA-binding helix-turn-helix motifs [58]. It has been predicted that these cysteine residues bind to metals via formation of metal-thiolate bonds and thus inhibit binding via the adjacent helix-turn-helix region [57,58]. Unlike ArsR/SmtB metalloregulatory proteins, above mentioned ZntR does not contain any cysteine residue and lacks the characteristic metal-binding elements [50]. However, it has two histidine-rich regions, one at the C-terminus and the other near the N-terminus. Similar histidine-rich regions have been reported for zinc and cobalt transporters which are thought to be domains for zinc-binding ions [36].

Metallothioneins (MTs) are known as proteins containing thiol groups in their structure, especially cysteine, which is the cause of affinity to metals—such as Cd, Pb, Hg, Cu or Zn [59,60,61]. The metallothionein gene, *smtA*, is controlled by the SmtB repressor [62], which also regulates a zinc-transporting P-type ATPase [56,57]. The primary function of MT is detoxification of heavy metals in living organisms, which became the subject of a number of studies [63,64,65]. Occurrence of metallothionein was observed in a variety of organisms and microorganisms such as bacteria, invertebrates or vertebrates [60,66,67,68,69].

In this study the presence of metallothionein in strains of *S. aureus* with resistance to the effects of heavy metals was determined. Much higher concentration of metallothionein (more than 6 µM) in RPb in comparison to the control has been reported and the other resistant strain RCd, where the concentration of metallothionein was much lower in comparison to the control *S. aureus*, values around 0.5 µM (Figure 5Ba). These results show possible variation in the metal resistance origin for cadmium and lead. While cadmium is mainly removed through the CadA transport system, lead is mainly removed after bounding to metallothioneins. CadA transport system was proofed as lead and cadmium transporter too [39]. Probably all the zinc/cadmium translocating P-type ATPases are also effective in lead ions export [39]. After the application of antibiotics the values of determined metallothionein in both resistant strains decreased, especially in RPb (Figure 5Bb). Only the application of streptomycin caused the growth of MT level in RCd compared to the control (*S. aureus*). Here, the presented results confirm the trends which are obvious in the evaluation of spectrophotometric analysis (Section 3.1.2.) and suggest that the mechanisms of cross resistance at ampicillin and streptomycin will be different because of various MT level in RCd. As it was mentioned above (Section 3.1.2.) the ampicillin and streptomycin caused cross resistance in RPb and here it is the confirmation of such deduction in the lowest MT levels for these combinations. It is necessary to remember that the presented results are related to the MT level inside of cells, therefore the higher change of MT level indicates the highest removal of complex MT-metal ions. The summarized view (Figure 5Bc) then shows a significant effect of antibiotic application, especially on RPb where the decrease of metallothionein is greatest. On the other hand, the application of the antibiotic caused an increase in the amount of metallothionein in RCd (Figure 5Bc). These results confirm the results obtained from the expression of *zntR* gene (Figure 5A) and thus confirm further the direct relationship between *zntR* gene and metallothionein expression. The great question is about mechanism of this relationship because till now no explanation was published about the influence of *zntR* gene expression on metallothionein level. Because of the fact that metallothionein production is closely connected with zinc level (zinc-sensing transcriptional repressor SmtB) [62], and *zntR* gene expression too [36], and both genes are chromosomally located, we only assumed the creation of similar effect which was called in the cross resistance formation as co-resistance. The second possibility, and it is important to highlight that it is only in hypothesis, is the reaction of *zntR* receptor for the presence of other metal ions (lead and cadmium) but this assumption was not proofed elsewhere.

The content of heavy metals in the samples was determined by atomic absorption spectrometry measurements. The determined content of metal ions is presented in Figure 5C. The concentrations of cadmium and lead ions (950 µM) in both samples were detected in almost total applied concentration (940 µM). The same concentration was measured in samples after the application of antibiotics. In control strain, the concentration of metals was below the detection limit (Figure 5C).

The influence of metallothioneins in the metal resistance was tested in some bacteria strains [70]. It must be said that the lead and cadmium resistance (in combination with antibiotics) in point of view of various resistance mechanisms (efflux, metallothioneins) are very seldom tested and there is no integrated output model of *S. aureus* resistance to these agents.

## 4. Conclusions

The reported experiments were performed to study the effects of antibiotic drugs (ampicillin, streptomycin, penicillin and tetracycline) on non-resistant strains of *S. aureus* and *S. aureus* strains resistant to the effects of heavy metal ions (cadmium or lead). Our results pointed to a significant antimicrobial effect of penicillin and tetracycline in both the control strain and the strains resistant to heavy metal ions. Microscopic methods only confirmed the morphological changes of resistant strains in comparison with the control, independently of the application of the tested antibiotic drug. On the other hand, the lead resistant *S. aureus* strain showed resistance to the effect of ampicillin and streptomycin. Cross resistance was thus observed only in RPb after the application of these two antibiotics. The obtained results can be used for further experiments with bacterial strains in terms of a deeper understanding of bacterial resistance caused by environmental factors.
