# Correction: Chudobova et al. Effect of Ampicillin, Streptomycin, Penicillin and Tetracycline on Metal Resistant and Non-Resistant *Staphylococcus aureus. Int. J. Environ. Res. Public Health* 2014, *11*, 3233–3255

**DOI:** 10.3390/ijerph22050744

**Published:** 2025-05-09

**Authors:** Dagmar Chudobova, Simona Dostalova, Iva Blazkova, Petr Michalek, Branislav Ruttkay-Nedecky, Matej Sklenar, Lukas Nejdl, Jiri Kudr, Jaromir Gumulec, Katerina Tmejova, Marie Konecna, Marketa Vaculovicova, David Hynek, Michal Masarik, Jindrich Kynicky, Rene Kizek, Vojtech Adam

**Affiliations:** 1 Department of Chemistry and Biochemistry, Faculty of Agronomy, Mendel University in Brno, Zemedelska 1, Brno CZ-613 00, Czech Republic; dagmar.chudobova@centrum.cz (D.C.); petrmichalek85@gmail.com (P.M.);; 2 Central European Institute of Technology, Brno University of Technology, Technicka 3058/10, Brno CZ-616 00, Czech Republic; 3 Department of Pathological Physiology, Faculty of Medicine, Masaryk University, Komenskeho namesti 2, Brno CZ-662 43, Czech Republic; 4 Karel Englis College, Sujanovo nam. 356/1, Brno CZ-602 00, Czech Republic

There was an error in the original publication [1]. We were notified that in Figure 5A, we inadvertently used the wrong working version of agarose gel that remained in the article throughout the pre-peer review and peer review process. We were able to immediately locate the correct datasets. The corrected figure, as well as the specific part of the Results and Discussion, can be found below:

The fluorescence intensity of the amplified fragment varies in dependence on the antibiotic type. In the RPb strains, the *zntR* gene’s expression was higher than that of control *S. aureus*. In contrast, in the RCd, the expression of the *zntR* gene was similar to the control *S. aureus* strain. In the RCd strain, after the addition of AMP and TTC, the expression of the *zntR* gene was significantly increased compared to the RCd without antibiotics. The most significant increase was observed after the application of ampicillin (Figure 5A(a)). The highest expression of the *zntR* gene was observed in the RPb strain without any addition of antibiotics. After the addition of antibiotics, the intensity of the expression was reduced (Figure 5A(a)). The expression of the *16S* gene (always present in bacteria) confirmed the presence of bacteria strain in the samples. This presence was independent of both on applied metal and antibiotics; therefore, the fluorescence intensity was constant (Figure 5A(b)).

**Figure 5:**
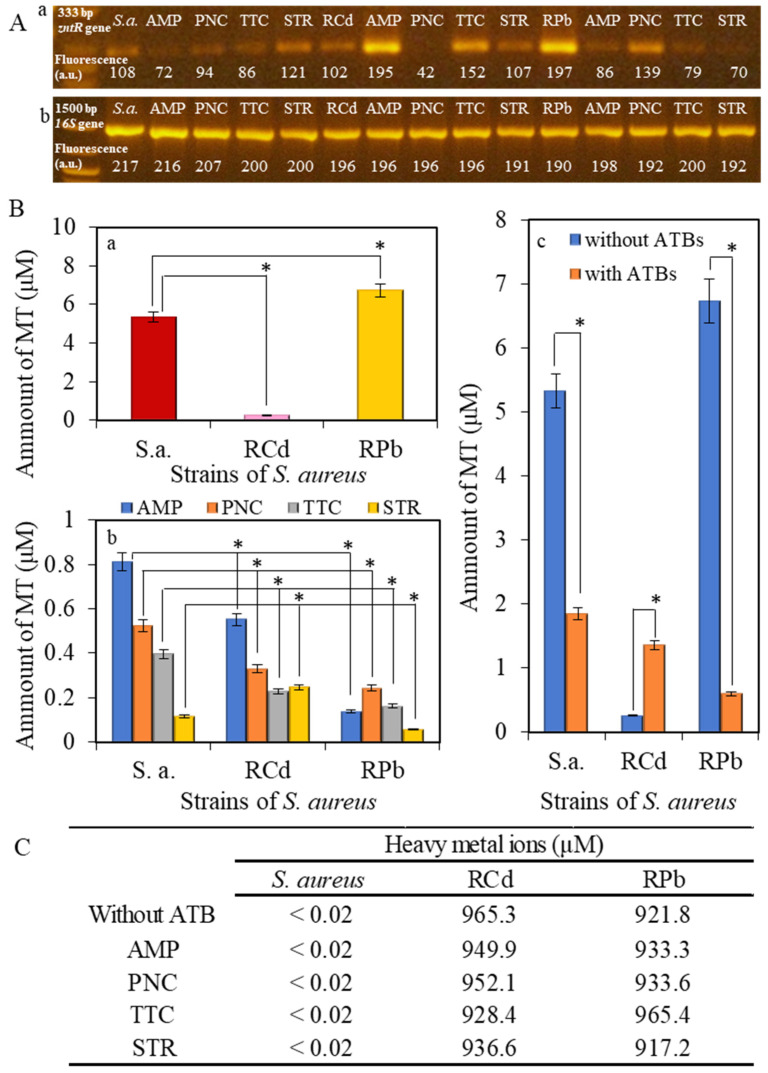
Electrochemical analysis of the non-resistant *S. aureus* or RCd or RPb: (**A**) gene expression with and without the application of antibiotics, (**a**) expression of *zntR* gene, (**b**) expression of 16S gene. (**B**) Determination of the amount of metallothionein (MT) in (**a**) *S. aureus*, RCd or RPb; and (**b**) *S. aureus*, RCd or RPb after application of 50 µM concentration of antibiotics; (**c**) comparison of the values of metallothionein in strains without application and after application of antibiotics. Presented values for ATBs are the sum of individual values for every antibiotic. All data represent mean ± s.d. NS, not significant, * *p* < 0.05, (**C**) Comparison of applied concentration of heavy metal ions (950 µM) with measured concentration using atomic absorption spectrometry.

The authors state that the scientific conclusions are unaffected. This correction was approved by the Academic Editor. The original publication has also been updated.
